# Long-term outcomes and predictors of percutaneous radiofrequency thermocoagulation of Gasserian ganglion for maxillary trigeminal neuralgia: a retrospective analysis of 1070 patients with minimum 2-year follow-up

**DOI:** 10.1080/07853890.2022.2117409

**Published:** 2022-09-23

**Authors:** Wenxing Zhao, Liqiang Yang, Ansong Deng, Zongjie Chen, Liangliang He

**Affiliations:** a Department of Pain Management, Xuanwu Hospital, Capital Medical University, Beijing, PR China; b Department of Anesthesiology and Pain, The First People’s Hospital of Chongqing Liang Jiang New Area, Chongqing, PR China

**Keywords:** Maxillary trigeminal neuralgia, radiofrequency thermocoagulation, pain-free survival, facial numbness, ophthalmic complications, masseter weakness

## Abstract

**Objective:**

To estimate long-term efficacy and safety for maxillary trigeminal neuralgia (TN) using radiofrequency thermocoagulation (RFT) targeted on Gasserian ganglion, and to identify the factors which may influence outcomes after procedure.

**Methods:**

From 2006 to 2019, 1070 patients underwent RFT for the treatment of medically refractory maxillary TN was included. All patients were followed up for at least 2 years. Outcomes and complications were recorded and analysed. Logistic regression analysis was employed to identify risk factors of long-term pain recurrence. Prognostic value was calculated from receiver-operating characteristic curve (ROC).

**Results:**

Longitudinal analysis was taken place for 97 non-responders (9.1%) with ineffective pain relief, 253 responders (23.6%) with pain recurrence and 720 responders (67.3%) without pain recurrence. The median pain-free survival (PFS) was 112.0 months (95% CI: 107.5, 116.5). The pain-free rates were 89.9% (95% CI: 88.0–91.8%) at 1 year, 83.8% (95% CI: 81.5–86.1%) at 2 years, 75.4% (95% CI: 72.7–78.1%) at 5 years and 70.2% (95% CI: 67.4–73.0%) at 10 years. Atypical facial pain (HR = 5.373, 95% CI: 2.623–11.004, *p* < .001), previous facial numbness (HR = 5.224, 95% CI: 3.107–8.784, *p* < .001) and poor initial response to medication (HR = 3.185, 95% CI: 2.087–4.860, *p* < .001) were independently associated with long-term pain recurrence. Patients with prognostic index (PI) > 0.25 were identified as high-risk for recurrent TN (HR = 5.575, 95% CI: 3.991–7.788, *p* < .001). New and worsen facial hypoesthesia was recorded in 77.9% of patients corresponding with BNI score II–IV, and 18.7% reported improved sensation. Severe complication incidence including troublesome dysesthaesia, keratitis and masseter weakness was higher in 80 °C group.

**Conclusions:**

Favourable outcomes were achieved in terms of long-term pain relief and complications rate after RFT for maxillary TN. Patients with typical facial pain, normal facial sensation, and good initial response to medications may have favourable long-term outcomes.

**Key messages**
- This is a retrospective analysis of radiofrequency thermocoagulation (RFT) targeted on Gasserian ganglion for the treatment of maxillary trigeminal neuralgia (TN) during long-term follow-up. Recurrence-free survival among a large sample was assessed and risk factors associated with long-term pain recurrence was identified. It has been verified that inadvertent damage of ophthalmic and mandibular division causes ophthalmic and masticatory complications. Therefore, a more precise needle tip position and thermocoagulation using a relatively low temperature was recommended.

## Introduction

Trigeminal neuralgia (TN) is a disorder characterized by recurrent episodes of severe facial pain in the distribution of trigeminal nerve with a crude annual prevalence of 4–13 cases per 100,000 people. And the incidence rate progressively increases with age from 17.5/100,000/year between 60 and 69 years of age up to 25.6/100,000/year after age 70 [1]. A subgroup of patients diagnosed of any category of TN including classic, secondary and idiopathic TN also surfer from concomitant continuous pain or back pain between the typically paroxysmal attacks, TN with the abovementioned presence of untypical facial pain has been defined as uncommon type of TN by the International Headache Society Classification (ICHD) [2]. Central facilitation of trigeminal nociceptive processing and progressive root damage have been proposed as the probably underlying mechanism of untypical pain [3,4]. The maxillary and mandibular branches are most affected, while the ophthalmic division is affected in <5% [5]. First line treatment for TN is the pharmacological therapy [6]. According to the newly published guidelines, neurosurgical interventions should be considered for TN patients who were refractory to medical treatment or were suffering disabling side effects related to high dosages of antiepileptic drugs [7]. Microvascular decompression (MVD) is generally recognized as the most effective technique for classic TN with morphological changes of neurovascular conflict (NVC), however, the risk of craniotomy and anaesthesia are still controversial especially for the elderly [8]. As an option of the percutaneous trans-foramen ovale (FO) techniques of Gasserian ganglion, radiofrequency thermocoagulation (RFT) is a minimally invasive procedure which is preferred to patients who are unfit for MVD and patients with recurrent pain after MVD or previous destructive procedures. It provides an approximately 80% chance of being pain-free at 1 year after treatment, with a relatively low rate of long-term pain recurrence [9]. Nonetheless, RFT is also accompanied by a chance of thermocoagulation complications including facial numbness, decreased corneal reflex and masseter weakness, which not only reduce patients’ satisfaction but also decrease patients’ quality of life [10–12]. Moreover, it has been verified with clinical trials that the technical difficulty of precise damage to the restrained distribution of V_2_ and the limited ability to assess real-time damage effect intraoperatively may induce ophthalmic complications and masseter weakness when targeting on maxillary division through FO route [13]. Therefore, the aim of this study was to estimate the long-term outcomes of RFT targeted on Gasserian ganglion for medically refractory maxillary TN, and identify the predictors of satisfactory outcome associated with surgical parameters and patient special characteristics in order to provide a helpful and clear contribution to patient selection and surgical technique standardization.

## Materials and methods

### Study design and patient sample

The protocol was reviewed and approved prior to study commencement by the Institutional Ethics Committee of Human Research of the First People’s Hospital of Chongqing Liang Jiang New Area in China. The written informed consent for the use of their data for future studies and publications was obtained from all the patients in the study. We retrospectively analysed data of 1070 patients who were submitted to percutaneous radiofrequency thermocoagulation (PT-RFT) of the Gasserian ganglion for treatment of medically refractory maxillary TN in the Department of Anaesthesiology and Pain at our institution from 1 January 2006 to 31 December 2019. Follow-up data were extracted from our prospectively maintained database. Clinical data were collected from the electronic medical records (EMR).

All the patients were recruited on the following criteria: (1) classical trigeminal neuralgia (CTN) of maxillary division (V_2_) but not suitable for MVD^2^; (2) idiopathic trigeminal neuralgia (ITN) of V_2_^2^, which was predefined as classical symptom of neuralgia in maxillary division without abnormality on radiological examination and any impairment in trigeminal somatosensory evoked potential; (3) neurosurgical intervention is needed according to guideline [14]; (4) complete clinical record data; (5) follow-up using the specific criterion; (6) the minimum follow-up was 2 years. Patients were excluded from the study if they did not fulfil the inclusion criteria (Table 1).

**Table 1.** Cohort selection criteria for patients with maxillary TN receiving RFT.

Exclusion criteria	Exclusions	Remaining	%
Assessed for eligibility	–	1705	100.00
Diagnosis of STN	287	1418	83.17
Un-sufficient medical record data	71	1347	79.00
Follow-up not using the specific criterion	135	1212	71.09
Follow-up duration <2 years	142	1070	62.76

TN: trigeminal neuralgia; STN: secondary trigeminal neuralgia; RFT: radiofrequency thermocoagulation

### Follow-up

Follow-ups according to the specific criterion were taken place on schedule by specially trained investigators who were not involved in the study. Patients were admitted for 2 d post RFT procedure, and initial facial pain outcome was assessed at discharge day by pain surgeons. Long-term follow-ups were carried out every 3 months for the first 2 year and thereafter every 6 months through telephone or email interviews. The collecting data were registered and preserved in the prospective database.

### Data extraction

Demographic and clinical data were comprised age, gender, co-morbidity, disease duration, affected side, pain distribution, baseline pain score, type of facial pain, MRI/MRA imaging, neurosurgical intervention history, facial hypaesthesia at baseline, initial improvement in response to medication and thermocoagulation temperature. These abovementioned data were extracted from our EMR. The long-term follow-up data encompassed the following parameters: pain-free interval in patients with pain recurrence, pain-free follow-up period in patients with pain relief, and complications such as facial hypaesthesia, masseter weakness, decreased corneal reflex, keratitis and ptosis.

### Definitions of outcome measures

Pain intensity was assessed using the numeric rating scale (NRS) which consists of 0–10 pain (0 = no pain, 1–3 = mild pain, 4–6 = moderate pain, 7–10 = severe pain). The Barrow Neurological Institute (BNI) scale was used to estimate the degree of pain after RFT [15]. If the BNI pain score was Class III–V, the treatment was considered as a failure. Hence, the initial effective rate (%) was pre-defined as [(BNI Class I + II)/*n*] × 100% at discharge day assessment. Pain recurrence was pre-defined as change from BNI Class I or II to a lower-outcome class (BNI Class III–V). Pain-free survival (PFS) was defined as the period of maintaining effective pain relief without medication from RFT procedure to the date of TN recurrence. BNI facial hypaesthesia scale was used to assess the degree of ipsilateral facial numbness [16]. Class I: no facial numbness; Class II: mild facial numbness and not bothersome; Class III: facial numbness and somewhat bothersome; Class IV: facial numbness and very bothersome (troublesome dysesthaesia).

The primary study endpoint for this study was PFS over long-term follow-up period. For patients with bilateral pain, only their primarily unilateral RFT in the more symptomatic side was included. To avoid repeat measurements, for patients who underwent RFT for TN in the same division for several times in our hospital, only the first RFT procedure was included.

### Procedure

**Figure 1.**
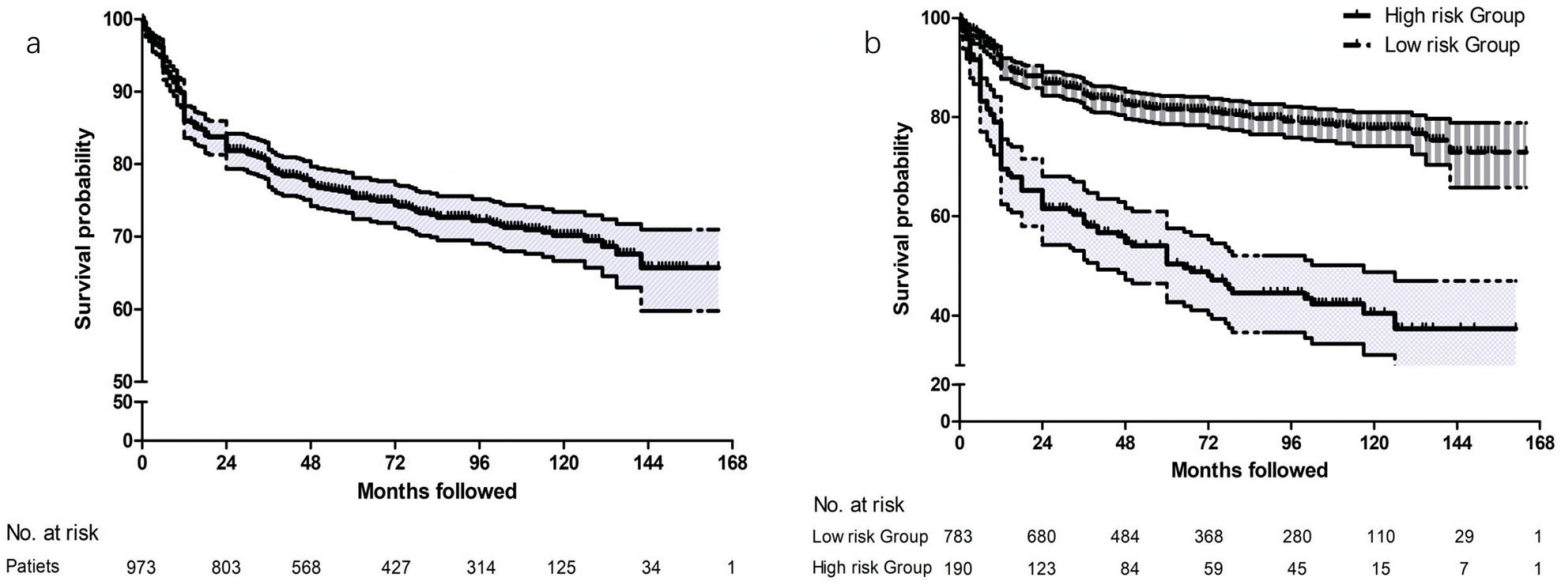
Puncture of Gasserian ganglion for on V2 selective RFT through FO approach. (a) The puncture needle entering the right FO along a quarter of the inner side wall of FO on the coaxial image of FL. (b) The tip of the needle is placed in middle third between the clivus and skull base on the lateral image of FL. V2: maxillary nerve; FO: foramen ovale; FL: fluoroscopy.

All RFT procedures for the treatment of maxillary division TN were performed in the operating room under fluoroscopic guidance by the same skilled pain surgeon, who demonstrated expertise in performing neurosurgical interventions of the Gasserian ganglion. Atropine (0.01 mg/kg), fentanyl (50 μg) and droperidol (2.5 mg) were administered 1 h before procedure. Electrocardiogram, blood pressure, and pulse oximetry were monitored. Patients lied in the supine position with the head in a reverse occipitomental position. The FO was visualized by rotating C-arm with 15–25 degrees in the coronal plane and 20–30 degrees in the sagittal plane. After sterilization and local infiltration of 10 mL 0.5% lidocaine, a 22-gauge, 15 cm radiofrequency needle with a 5 mm active tip (Cosman TIC-C5 electrode; Cosman Medical, Burlington, MA) was advanced directly towards FO using the Hartel anterior approach. Once the tip of the needle reached approximately 3 mm into FO, examinations were repeatedly conducted under the guidance of fluoroscopy in coaxial and lateral view. The location of the needle tip should be confirmed to be in the middle third between the clivus and the skull base in the lateral images (Figure 1). Multiple adjustments were conducted to correct the precise position according to patients’ response to sensory (50 Hz, 1 ms) and motor (2 Hz, 0.1 ms) stimulation test [17]. If patient’s subjective report of paraesthesia and/or twitching in response to <0.1 V, 0.1–0.3 V and 0.3–0.5 V sensory stimulation in the affected maxillary dermatome, the parameters of RFT were set to 70 °C, 75 °C and 80 °C for 90 s, respectively. All patients were sedated by intravenous propofol at a dose of 1.5–2.0 mg/kg, which could be supplemented (0.05 mg/kg) according to the depth of anaesthesia during thermocoagulation [17].

### Statistical analysis

All statistical analyses were conducted using SPSS for version of 22.0 (SPSS Inc., Chicago, IL). Differences were considered statistically significant at the 5% level with 2-tailed test.

Quantitative data were described in the median with interquartile range (IQR). Categorical variables were reported in frequencies and proportions, and compared with Chi-square test or Wilcoxon rank sum test. The Kaplan–Meier curves with 95% confidence interval (CI) were calculated to explore the probability of PFS. The censored point was considered as pain-free surviving at the last follow-up and at the last contact. For patients who were lost to follow-up or patients who died of other diseases, data obtained at the time of the last contact was used. Univariate regression analysis and multivariate analysis based on binary logistic regression was used to identify and predict independent risk factors for pain recurrence in the long-term follow-up. The receiver-operating characteristic curve (ROC) was employed to determine the optimum cut-off value of prognostic index (PI) to discriminate patients with high and low risk for recurrent TN. Differences between PI-dependent survival were compared using the log-rank test.

## Results

### Characteristics of the study patients

This study comprised a total of 1705 patients receiving RFT for medically refractory maxillary TN. A cohort of 1070 patients (62.76%) met the inclusion criteria (Table 1). Longitudinal analysis was taken place for 97 non-responders (9.1%) with ineffective pain relief at discharge-day assessment, 253 responders (23.6%) with pain recurrence and 720 responders (67.3%) without pain recurrence.

Baseline characteristics were not balance between patients with effective and ineffective initial pain relief in Table 2. The rate of atypical facial pain was 4.6% in patients who experienced effective initial outcome, whereas it was 9.3% among patients who reported Class III–V according to BNI pain score (*p* = .046). Before RFT procedure, facial hypaesthesia existed in 6.2% of patients with immediate effective pain relief, and 12.4% of them was noted in patients without satisfactory immediate pain relief preoperatively (*p* = .020).

**Table 2.** Demographic characteristics of patients with and without initial effective response.

Variables	BNI score 1–2	BNI score 3–5	*p* Value
	(*n* = 973)	(*n* = 97)	
Age-year (median, range)	62 (17–91)	64 (32–83)	.109
Gender-no. (%)			.450
Male	383 (39.4%)	42 (43.3%)	
Female	590 (60.6%)	55 (56.7%)	
Co-morbidity-no. (%)			.761
Hypertension	121 (17.0%)	14 (14.4%)	
Diabetes mellitus	272 (38.1%)	40 (41.2%)	
Disease duration-mo (median, range)	60 (2–600)	48 (3–360)	.923
Baseline NRS score (median, range)	8 (4–10)	8 (5–10)	.662
Affected side-no. (%)			.428
Left	391 (40.2%)	43 (44.3%)	
Right	570 (58.6%)	54 (55.7%)	
Bilateral	12 (1.2%)	0 (0%)	
Type of facial pain-no. (%)			.046
Recurrent paroxysmal pain	928 (95.4%)	88 (90.7%)	
Atypical pain	45 (4.6%)	9 (9.3%)	
MRI/MRA pre-RFT-no. (%)			.100
Normal	883 (90.8%)	83 (85.6%)	
Neurovascular conflict	90 (9.2%)	14 (14.4%)	
History of previous neurosurgery-no. (%)			.222
Previous MVD	25 (2.6%)	3 (3.1%)	
Previous RFT	104 (10.7%)	10 (10.3%)	
Previous ablative neurosurgery	44 (4.5%)	9 (9.3%)	
Facial hypaesthesia pre-RFT-no. (%)			.020
Normal	913 (93.8%)	85 (87.6%)	
Altered facial sensation	60 (6.2%)	12 (12.4%)	
Initial improvement in response to medication			.065
No improvement	109 (11.2%)	17 (17.5%)	
Initial improvement	864 (88.8%)	80 (82.5%)	
RFT temperature-no. (%)			.413
70 °C	390 (40.1%)	41 (42.3%)	
75 °C	487 (50.1%)	43 (44.3%)	
80 °C	96 (9.9%)	13 (13.4%)	
Follow-up duration-mo (median, range)	97 (24, 178)	115 (25, 161)	<.001

NRS: numeric rating scale; BNI: Barrow Neurological Institute; MVD: microvascular decompression; RFT: radiofrequency thermocoagulation; MRI: magnetic resonance imaging; MRA: magnetic resonance angiography

### Efficacy outcomes

**Figure 2.**
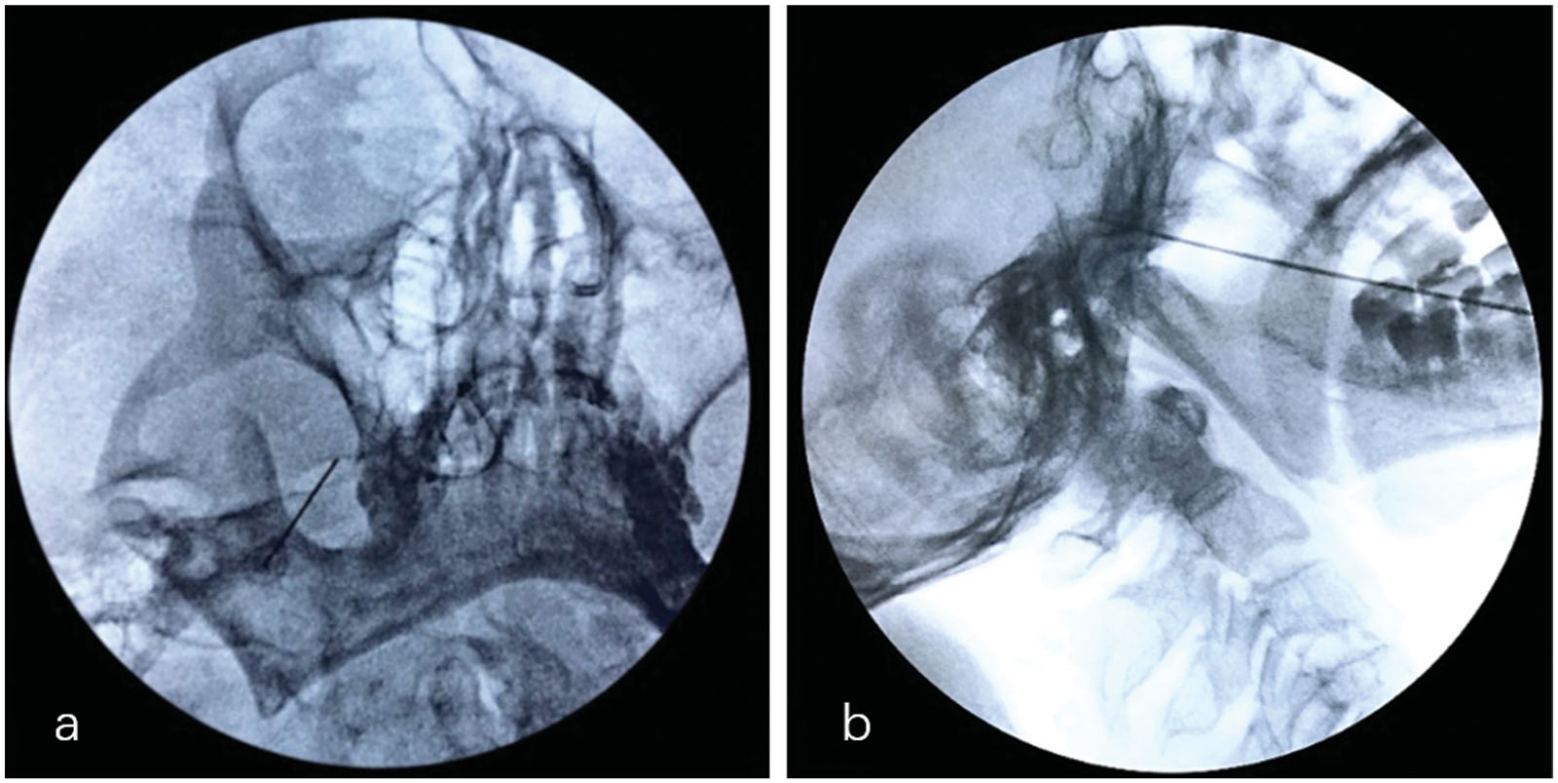
(a) Kaplan–Meier survival curve for patients with maxillary division TN after RFT over a 14-year follow-up period. The median pain-free survival was 112.0 months (95% CI: 107.5, 116.5). Tick marks illustrated censored observations. (b) Kaplan–Meier survival curves for patients in high-risk group and low-risk group after RFT over a 14-year follow-up period. Patients with PI > 0.387 had a higher risk for pain recurrence with HR = 5.575 (95% CI: 3.991–7.788, *p* < .001). Tick marks illustrated censored observations. TN: trigeminal neuralgia; RFT: radiofrequency thermocoagulation; CI: confidence interval; PI: prognostic index.

Figure 2(a) illustrates the cumulative survival curve of patients. The median follow-up duration was 100 (IQR: 32, 168) (range: 24, 178) months. The median PFS was 112.0 months (95% CI: 107.5, 116.5). The pain-free rates were 89.9% (95% CI: 88.0–91.8%) at 1 year, 83.8% (95% CI: 81.5–86.1%) at 2 years, 75.4% (95% CI: 72.7–78.1%) at 5 years and 70.2% (95% CI: 67.4–73.0%) at 10 years.

### Risk factors associated with pain recurrence after RFT for maxillary TN

Univariate analysis results are shown in Table 3. Before RFT procedure, an existence of atypical facial pain (HR = 6.277, 95% CI: 2.824–13.952, *p* < .001), facial hypaesthesia (HR = 6.696, 95% CI: 3.680–12.183, *p* < .001), and initially refractory response to medication (HR = 4.200, 95% CI: 2.632–6.765, *p* < .001) were significant risk factors associated with long-term pain recurrence. Additionally, no significant association with pain recurrence was observed on variables including age, gender, co-morbidity, disease duration, baseline NRS score, affected side, NVC on MRI/MRA, history of previous neurosurgical intervention, RFT temperature, NRS post-RFT at discharge day, initial efficacy, BNI facial hypaesthesia score post-RFT, ophthalmic complications post-RFT and masseter weakness post-RFT.

**Table 3.** Recurrence risk factors of the patients from univariate regression.

Variables	No. of patients	Hazard ratio (HR)	95% Confidence interval (CI)	*p* Value
Age (years)				
≤60-year old	455	–	–	–
>60-year old	615	1.107	0.799–1.533	.542
Gender				
Female	590	–	–	–
Male	383	1.150	0.829–1.595	.401
Co-morbidity	–	–	–	.323
None	623	–	–	–
Hypertension	135	1.115	0.920–1.375	.331
Diabetes mellitus	312	1.230	0.914–1.583	.245
Disease duration (months)				.316
<12 months (<1 year)	173	–	–	–
12–24 months (1–2 years)	121	1.100	0.575–2.104	.773
24–48 months (2–4 years)	216	1.605	0.938–2.744	.084
48–72 months (4–6 years)	136	1.723	0.962–3.085	.067
72–96 months (6–8 years)	92	0.972	0.469–2.016	.940
96–120 months (8–10 years)	130	1.272	0.679–2.380	.452
>120 months (10 years)	202	1.610	0.934–2.775	.086
Baseline NRS score				.054
Moderate pain	190			
Severe pain	763	1.876	1.180–2.984	.078
Complete pain	117	2.006	1.066–3.777	.061
Affected side				.828
Left	434			
Right	624	1.084	0.780–1.508	.630
Bilateral	12	1.405	0.306–6.442	.662
Type of facial pain				
Recurrent paroxysmal pain	1016			
Atypical pain	54	6.277	2.824–13.952	<.001
MRI/MRA pre-RFT				
Normal	966			
Neurovascular conflict	104	0.468	0.036–6.055	.561
History of previous neurosurgery				.105
None	875			
Previous MVD	28	0.665	0.254–1.741	.407
Previous RFT	114	1.565	1.018–2.406	.041
Previous ablative neurosurgery	53	1.452	0.748–2.689	.235
Facial hypaesthesia pre-RFT				
Normal	998	–	–	–
Altered facial sensation	72	6.696	3.680–12.183	<.001
Initial improvement in response to medication				
Initial improvement	948	–	–	–
No improvement	122	4.220	2.632–6.765	<.001
RFT temperature	–	–	–	.743
70 °C	617	–	–	–
75 °C	362	0.985	0.728–1.167	.861
80 °C	91	1.203	0.815–1.376	.491
NRS post-RFT at discharge	–	–	–	.190
No pain	841	–	–	–
Mild pain	142	0.529	0.314–0.892	.017
Moderate pain	42	1.549	0.306–7.841	.597
Severe pain	42	1.066	0.096–11.799	.959
Complete pain	3	0.471	0	1.000
Initial efficacy				
Immediate effective response	973	–	–	–
Immediate negative response	97	0	0	.995
BNI facial hypaesthesia score post-RFT	–	–	–	.818
Class I	237		–	–
Class II	564	0.912	0.604–1.377	.662
Class III	211	0.826	0.494–1.382	.466
Class IV	58	0.710	0.310–1.625	.417
Ophthalmic complications post-RFT				
No	995	–	–	–
Yes	75	0.498	0.243–1.021	.057
Masseter weakness post-RFT				
No	991	–	–	–
Yes	79	0.694	0.362–1.330	.271

MVD: microvascular decompression; RFT: radiofrequency thermocoagulation; NRS: numeric rating scale; BNI: Barrow Neurological Institute; MRI: magnetic resonance imaging; MRA: magnetic resonance angiography

In multivariate analysis, the hazard ration for long-term recurrence was 5.373 (95% CI: 2.623, 11.004) for atypical facial pain (*p* < .001), 5.224 (95% CI: 3.107, 8.784) for facial hypaesthesia assessed by BNI scale (*p* < .001), and 3.185 (95% CI: 2.087, 4.860) for no initial improvement in response to medication (*p* < .001). The contribution of all the other variables to prediction was trivial in this study (Table 4). The individual PI value could be calculated by the regression coefficients in the multivariate binary logistic regression model (sensitivity= 70.6%, specificity = 83.7%):

PI=−1.078+1.681×atypical facial pain (yes=1)+1.653×facial hypesthesia (yes=1)+1.15×initially refratory response to medication (yes=1)

**Table 4.** Recurrence risk factors of the patients from multivariable regression.

Independent variables	Regression coefficient		Adjusted hazard ratio			*p* Value
	B	SE	Exp (B)	95% CI		
				Lower	Upper	
Type of facial pain	1.681	0.366	5.373	2.623	11.004	<.001
Recurrent paroxysmal pain (0)						
Atypical pain (1)						
BNI facial hypaesthesia scale pre-RFT	1.653	0.265	5.224	3.107	8.784	<.001
Normal (0)						
Altered facial sensation (1)						
Initial improvement in response to medication	1.158	0.216	3.185	2.087	4.860	<.001
Initial improvement (0)						
No improvement (1)						

CI: confidence interval; BNI: Barrow Neurological Institute; RFT: radiofrequency thermocoagulation

The ROC analysis revealed that the Youden index value was 0.25. Accordingly, patients with PI > 0.387 (optimum cut-off value) were identified as high-risk for long-term pain recurrence (HR = 5.575, 95% CI: 3.991–7.788, *p* < .001). The median recurrence free survival of high-risk was significantly lower than that of low-risk group (57.33 (95% CI: 48.80–65.87) *vs.* 130.61 (95% CI: 126.08–135.13) (Figure 2(b)).

### Adverse events

Adverse events after RFT procedure are demonstrated in Table 5. According to BNI facial hypaesthesia scale, 237 patients (22.1%) reported no facial numbness after RFT procedure, 546 (52.7%), 211 (19.7%) and 58 (5.4%) patients, respectively, reported ipsilateral facial numbness of Class II, III, and IV (troublesome dysesthaesia). A total of 79 patients (7.4%) reported masseter weakness after RFT procedure. In addition, there were 75 patients (7.0%) reporting ophthalmic complications, such as decreased corneal reflex, keratitis and ptosis. Compared to 70 °C and 75 °C group, the incidence of troublesome dysesthaesia, ophthalmic complications and masseter weakness were significantly higher in 80 °C group. Concerning improvement of postoperative facial hypaesthesia, gradual improvement was reported in a total of 200 (18.7%) patients, and the figures were considerably higher in the 70 °C and 75 °C group than that in the 80 °C group (*p* = .004).

**Table 5.** Adverse event post-RFT of the patients.

Variables	RFT temperature			*p* Value	Total (*n* = 1070) (%)
	70 °C (*n* = 617) (%)	75 °C (*n* = 362) (%)	80 °C (*n* = 91) (%)		
BNI facial hypaesthesia scale after RFT-no. (%)				<.001	
Class I	137 (22.2)	95 (26.2)	5 (5.5)	–	237 (22.1)
Class II	397 (64.3)	133 (36.7)	34 (37.4)	–	564 (52.7)
Class III	57 (9.2)	115 (31.8)	39 (42.9)	–	211 (19.7)
Class IV	26 (4.2)	19 (5.2)	13 (14.3)	–	58 (5.4)
Facial numbness resolved during follow-up-no. (%)				.004	
Class II	71 (11.5)	57 (15.7)	8 (8.8)	–	136 (12.7)
Class III	36 (5.8)	23 (6.4)	5 (5.5)	–	64 (6.0)
Class IV	0	0	0	–	0
Ophthalmic complications -no. (%)	41 (6.6)	27 (7.5)	19 (20.9)	<.001	75 (7.0)
Masseter weakness -no. (%)	42 (6.8)	22 (6.1)	15 (16.5)	.002	79 (7.4)

RFT: radiofrequency thermocoagulation; BNI: Barrow Neurological Institute

## Discussion

In this study, there was a sufficient sample size to analyse the long-term outcomes and influence of various factors including patient special characteristics and surgical parameters on the recurrence after RFT of Gasserian ganglion for the treatment of maxillary division TN. We showed that the recurrent rate was approximately 23.6%. The predictive model identified three risk factors including atypical facial pain, facial hypaesthesia and initial refractory response to medication were associated with long-term pain recurrence. In addition, PI > 0.387 could lead to a relatively higher risk for recurrence. These results might provide convincing evidence of the long-term recurrent rate and risk factors associated with the impact of relapse.

Due to its relatively balanced efficacy and safety profile, percutaneous RFT of Gasserian ganglion through FO route has been extensively used to treat TN since 1974 [18]. It was able to achieve an immediate pain relief rate of approximately 85–97% for classical or idiopathic TN [11,19]. Consistent with previous studies, our results showed that a total of 973 cases (90.9%) obtained an effective pain relief immediately at the discharge day. The long-term outcomes of RFT for TN had been proposed in a number of previous clinical studies. Yuanzhang Tang et al. reported that excellent pain relief was 100% at discharge, 85% at 1 year, 75% at 3 years, 71% at 5 years and 49% at 10 years after CT-guided RFT of trigeminal ganglion [20]. A cohort study about the long-term effective rate of different branches of ITN after single RFT found that V_2_ division TN obtained the best excellent pain relief after RFT procedure with pain relief rate 91, 89, 80, 72, 60 and 54% at 1, 3, 5, 7, 9 and 11 years, respectively [21]. In our study, Kaplan–Meier curve of a large sample illustrated that the rate of pain-free without medication was respectively 89.9, 83.8, 75.4 and 70.2% at 1, 2, 5 and 10 years with a median PFS of 112.0 months (95% CI: 107.5, 116.5) (Figure 2(a)). Our findings indicated that RFT of Gasserrian ganglion for maxillary division TN provided a high rate of immediately pain relief, and satisfactory long-term efficacy could be expected during a long-term follow-up period. We supposed that the maxillary division could be ablated by a higher thermocoagulation temperature at 70–80 °C after confirmation of paraesthesia overlapping the restrained distribution of V_2_ without affecting the normal area due to its pure sensation conduction component.

Atypical facial pain was characterized more by constant aching or burning aspects, and could appear along with recurrent paroxysmal typical TN. There was the evidence that as opposed to typically paroxysmal pain, atypical pain consisting of continuous component might improve differently after MVD [22,23]. Additionally, several previous researches studied confirmed that it was an important factor leading to TN recurrence after neurosurgical intervention [17,24], which was also proven in our study. When a patient with atypical facial whose symptoms are usually incongruent with the more common aetiology was encountered, the poor immediate pain relief rate could be near 16.6% (*p* = .046) and an almost 5.373-fold increase in recurrent risk was estimated during long-term follow-up (95% CI: 2.623, 11.004, *p* < .001). Meanwhile, several previous studies found that a better long-term efficacy was observed in patients who had not undergone a previous neurosurgical intervention [18,25]. We speculated that the number of previous operations could not influence outcome, and initial failed treatment was not a factor affecting the pain control according to univariate analysis (*p* = .105). But the high recurrence rate could be increased by facial hypaesthesia which was mostly remaining side effects from the previous ablative neurosurgical interventions, such as RFT, PBC, SRS and peripheral neurectomy. In our study, patients with remained facial hypaesthesia were more likely to experience a failure immediately after RFT (*p* = .020), which might be explained by lack of a clear response to electrical stimulation tests. And these patients had a 5.224-fold increased risk for recurrence (95% CI: 3.107, 8.784, *p* < .001) according to the multivariable analysis. For parts of these patients, their TSEP results before RFT procedure in this study illustrated a prolonged latency of P1 and N2 or a reduced amplitude of some waveforms. Results were better in patients with good initial response to medication, patients whose medication had initially failed had a 3.185-fold increased risk for recurrence over the long-term follow-up (95% CI: 2.087, 4.860, *p* < .001). By ROC analysis, patients were discriminated into high-risk group for recurrence with PI > 0.387, they had a 5.575-fold increase in recurrent risk (95% CI: 3.991, 7.788, *p* < .001). The prediction model with sensitivity= 70.6% and specificity = 83.7% could be expected to provide a helpful and clear contribution to decision-making in the routine clinical patients selection before RFT procedure.

Heat could be produced by vibration and friction of radiofrequency electricity leading to thermocoagulation and denaturation of targeted nerve. Hence, various complications related to thermocoagulation could be developed and prevent the widespread use of RFT [26]. An observational study estimated the effectiveness and safety of RFT of the V_1_ (10%), V_1_ + V_2_ (63.7%, and V_1_ + V_2_ + V_3_ (26.3%) of TN. 97.5% patients experienced tolerable numbness, 17.5% patients experienced mildly decreased corneal reflex, 2.5% patients felt a foreign body sensation [27]. As our results showed, there were no surgical puncture-related complications after RFT procedure. 237 patients (22.1%) reported no facial numbness after RFT procedure, 546 (52.7%), 211 (19.7%) and 58 (5.4%) patients, respectively, reported ipsilateral facial numbness of Class II, III and IV (troublesome dysesthaesia). A recent research assessing long-term efficacy and complications of RFT at different temperatures for TN concluded that the long-term analgesic effects of RFT at high temperatures (≥80 °C) are not superior to those at relatively low temperatures (60–75 °C). In contrast, higher temperature has indeed been related to increased postoperative complications, especially in the form of facial numbness, masticatory muscles weakness and corneal hypoesthesia [10]. In our research, thermocoagulation temperature was not a significant factor for predicting PFS, but adverse events after RFT procedure were related to thermocoagulation temperature during the procedure. Compared to 70 and 75 °C group, the incidence of troublesome dysesthaesia, ophthalmic complications and masseter weakness were significantly higher in 80 °C group. Bing Huang et al. developed a novel CT-guided technique to block the V2 through foramen rotundum (FR), they found this new technique had the same good immediate and sustained pain relief as the conventional FO approach, and had a better adverse events profile associated with non-specific ablation in V1 and V3 dermatomes [28]. Recently, a comparative study also compared RFT through FR *versus* FO for the treatment of V_2_ TN and reported that the incidences of facial numbness and swelling did not differ significantly between the 2 groups (all *p* > .05). There was no postoperative corneal involvement or masticatory weakness in the FR group. However, corneal involvement and masticatory weakness occurred postoperatively in 55% patients and 77.5% patients in the FO group. They were unable to avoid the V_1_ and V_3_ branches, despite multiple adjustments of the needle position in FO group in 87.5% patients [29]. According to our results, a total of 79 patients (7.4%) reported masseter weakness after RFT procedure. In addition, there were 75 patients (7.0%) reporting ophthalmic complications, such as decreased corneal reflex, keratitis and ptosis. To explain the abovementioned results, we hypothesized that facial numbness, ophthalmic complication and masseter weakness primarily related to thermocoagulation heat, but imprecise position of radiofrequency trocar tip was also the mainly underlying reason when performing RFT procedure for treatment of maxillary division TN [30]. To decrease these complications, we suggested a more precise needle tip position and thermocoagulation using a relatively low temperature as the key to safe RFT and satisfactory PFS with fewer complications. To our knowledge, temperature range from 70 to 75 °C for the treatment of V_2_ or V_3_ TN could effectively ablate the conduction of pain fibres and spare the motor nerve. Generally, in this study, the needle tip was precisely adjusted according to the patients’ response to electrical stimulation tests after verification *via* fluoroscopic imaging. Multiple electrical stimulation tests were needed to elicit paraesthesia in the maxillary dermatome, while minimizing it in the dermatome of ophthalmic division and mandibular division. If paraesthesia was elicited in the affected V_2_ dermatome at > 0.5V, the position of the trocar tip should be continuously adjusted. If rhythmic myoclonus was elicited in mandible angle that indicated the needle tip was at motor fibre of V_3_, thus the position should also be adjusted. If paraesthesia could be elicited by <0.1 V sensory stimulation in the restrained V_2_ dermatome, patients would receive RFT at 70 °C for 90 s. If paraesthesia could be elicited by 0.1–0.3V sensory stimulation in the restrained V2 dermatome, patients would receive RFT at 75 °C for 90 s. During the thermocoagulation, patients should be repeatedly awakened for confirmation of the needle tip position according to appropriate response to intra-op electrostimulation. Additionally, corneal reflex and masseter function should be carefully monitored. If the ipsilateral corneal reflex was decreased which demonstrated V_1_ was being damaged, the procedure should be immediately halted. If the pain corresponding to the affected division disappeared completely and the tactile sense was blunt, the needle should also be pulled out. After RFT for maxillary TN, corneal reflex test should be taken place for each patient at discharge day. If there were decreased corneal reflex or other uncomfortable eye symptoms, ophthalmology consultation was immediately recommended for patients to avoid keratitis or permanent blindness eventually. In conclusion, ophthalmic complications and masseter weakness after RFT procedure of Gasserian ganglion for maxillary TN should be seriously taken into consideration, the above-mentioned protocol suggesting a more precise needle tip position and thermocoagulation using a relatively low temperature was recommended in purse of long-term pain relief as well as low risk for ophthalmic complications and masseter weakness after thermocoagulation.

There were several limitations in this study. First, undetected confounders and probable bias were inevitable problems due to the retrospective analysis with observational data. Second, participants bias including different mood, possible suggestibility and hearing loss might be carried while conducting phone or e-mail interview. However, potential methods of prevention, such as indirect, craft open-ended questions and neutral stance were not used in the study. Third, the follow up was not by an independent observer, this oversight might bias the results of the study. Future large-scale, long-term randomized studies would be needed to validate our findings.

## Conclusion

Our results indicated that patients would attain a long-term pain-free after RFT of Gasserian ganglion for the treatment of maxillary division TN. The predictive model identified three risk factors including atypical facial pain, facial hypaesthesia and initial refractory response to medication were associated with long-term pain recurrence. Additionally, more precise and accurate position of radiofrequency trocar tip in the restrained distribution of V2 division was necessarily required in order to decrease the occurrence of ophthalmic and masticatory complication.

## Data Availability

The data that support the findings of this study are available from the corresponding author, Professor Liangliang He, upon reasonable request.

## Abbreviations

TN: trigeminal neuralgia
CTN: classical trigeminal neuralgia
STN: secondary trigeminal neuralgia
ITN: idiopathic trigeminal neuralgia
RFT: radiofrequency thermocoagulation
FO: foramen ovale
FR: foramen rotundum
PFS: pain-free survival
MVD: microvascular decompression
MS: Multiple sclerosis
SRS: stereotactic radiosurgery
EMR: electronic medical records
BNI: Barrow Neurological Institute
NRS: numeric rating scale
IQR: interquartile range
CI: confidence interval
PI: prognostic index
CSF: cerebral spinal fluid.
